# Brain–lung interactions and mechanical ventilation in patients with isolated brain injury

**DOI:** 10.1186/s13054-021-03778-0

**Published:** 2021-10-13

**Authors:** Mairi Ziaka, Aristomenis Exadaktylos

**Affiliations:** 1 Department of Internal Medicine, Thun General Hospital, Thun, Switzerland; 2 Department of Emergency Medicine, Inselspital, University Hospital, University of Bern, Bern, Switzerland

**Keywords:** Mechanical ventilation, Acute respiratory distress syndrome, Ventilator induced lung injury, Brain damage, Brain–lung interactions, Inflammation

## Abstract

During the last decade, experimental and clinical studies have demonstrated that isolated acute brain injury (ABI) may cause severe dysfunction of peripheral extracranial organs and systems. Of all potential target organs and systems, the lung appears to be the most vulnerable to damage after brain injury (BI). The pathophysiology of these brain–lung interactions are complex and involve neurogenic pulmonary oedema, inflammation, neurodegeneration, neurotransmitters, immune suppression and dysfunction of the autonomic system. The systemic effects of inflammatory mediators in patients with BI create a systemic inflammatory environment that makes extracranial organs vulnerable to secondary procedures that enhance inflammation, such as mechanical ventilation (MV), surgery and infections. Indeed, previous studies have shown that in the presence of a systemic inflammatory environment, specific neurointensive care interventions—such as MV—may significantly contribute to the development of lung injury, regardless of the underlying mechanisms. Although current knowledge supports protective ventilation in patients with BI, it must be born in mind that ABI-related lung injury has distinct mechanisms that involve complex interactions between the brain and lungs. In this context, the role of extracerebral pathophysiology, especially in the lungs, has often been overlooked, as most physicians focus on intracranial injury and cerebral dysfunction. The present review aims to fill this gap by describing the pathophysiology of complications due to lung injuries in patients with a single ABI, and discusses the possible impact of MV in neurocritical care patients with normal lungs.

## Introduction

**Fig. 1:**
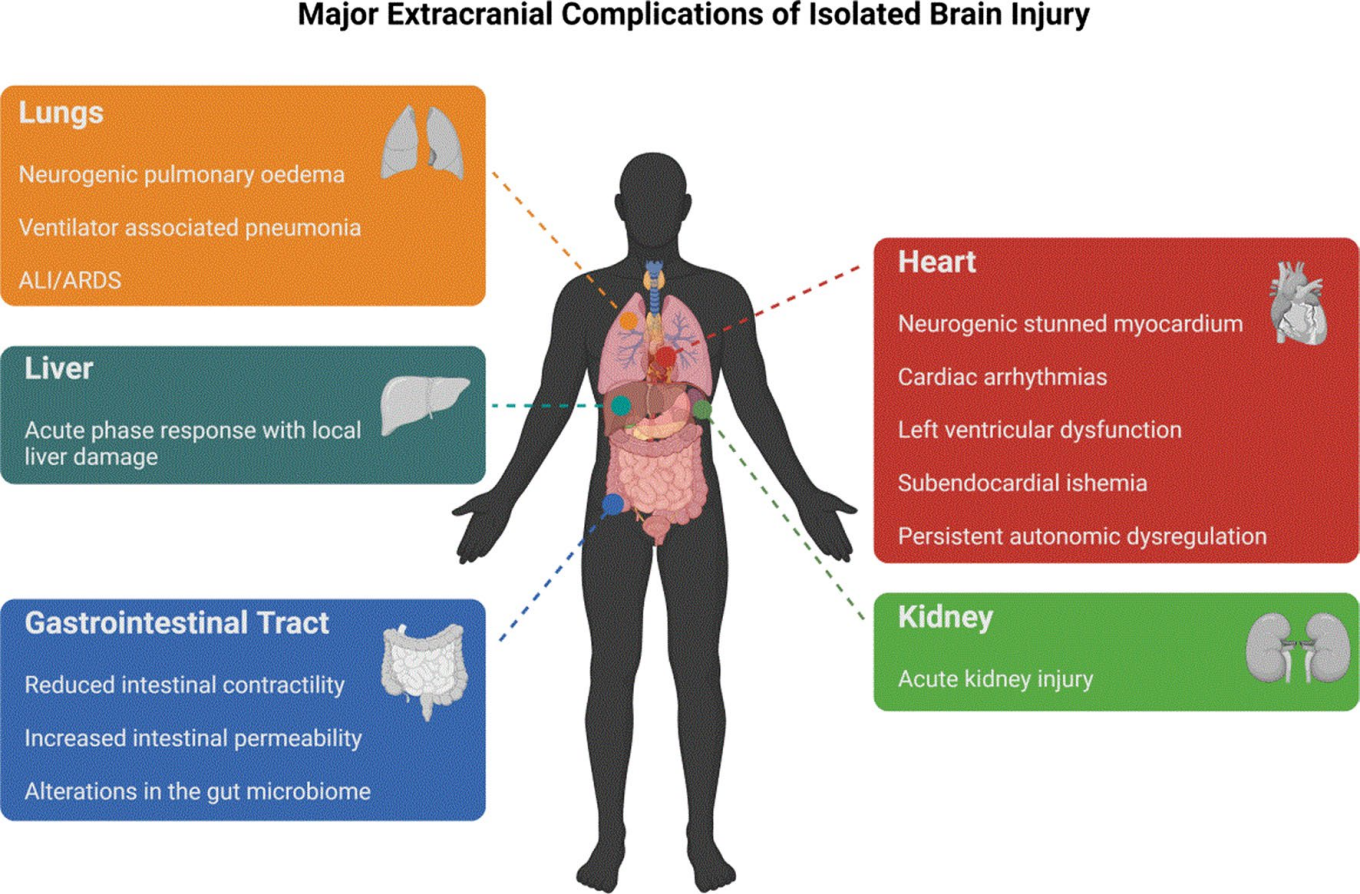
Major extracranial complications in patients with isolated acute brain injury. ALI, Acute Lung Injury; ARDS, Acute Respiratory Distress Syndrome

ABI, such as traumatic brain injury (TBI), intracerebral haemorrhage, subarachnoid haemorrhage (SAH), and acute ischemic stroke, is a serious public health problem; morbidity and mortality are high and survivors often experience extensive neurological disabilities [1, 2]. It has been shown that BI is not confined to the central nervous system [3], but may extend to distal organs and systems, and may ultimately lead to extracranial complications, including respiratory, cardiac, renal, lymphatic, and hepatic injuries [2, 4, 5] (Fig. 1). The pathophysiology of brain–lung interactions is complex and involves NPO, immune responses, inflammation, neurodegeneration, neurotransmitters, and dysfunction of the autonomic system [1, 6].

Of all potential target organs and systems, the lung appears to be the most vulnerable to damage after BI [2, 7]. Thoracic complications are highly prevalent in patients with ABI [8]. BI induces changes in the mechanics of the respiratory system, such as increased elastance and airway resistance [9], and leads to systemic and pulmonary inflammation, as well as increased pulmonary hydrostatic pressures and endothelial permeability [8]. On the other hand, the effects of systemic and pulmonary inflammation in patients with BI create a systemic inflammatory environment that makes lungs more vulnerable to secondary neurointensive care procedures that enhance inflammation, such as MV [7]. Indeed, MV in BI patients presents a number of unique challenges. Ventilator settings should be orientated to potential adverse cerebrovascular effects, the interactions of ventilation with intracranial circulation, cerebral autoregulatory reserve and brain compliance, in order to avoid intracranial hypertension and reduced cerebral blood flow (CBF) [10]. Unfortunately, the optimal ventilation strategy in BI patients with and without respiratory injury remains unclear. Although protective ventilation cannot be easily applied in BI patients, current knowledge suggests that it increases neurophysiological protection and seems to be preferable in critically ill patients with BI [11, 12].

The aim of the current review is twofold: firstly, to describe the pathophysiology of complications due to lung injuries in patients with a single ABI, and secondly, to discuss the possible impact of MV in neurocritical care patients with normal lungs.

## Lung injuries in acute brain damage

The most frequent pulmonary complications and the principle causes of acute respiratory failure in patients after BI include pneumonia associated with aspiration or the use of a ventilator, NPO, and acute respiratory distress syndrome (ARDS) [13].

### Ventilator-associated pneumonia

The incidence of ventilator-associated pneumonia in patients with ABI varies between 21 and 60% [14–16], with a pooled incidence of 36% [17].

Even though VAP is a common complication among ICU patients in general, patients with severe BI exhibit an inherently higher risk of VAP [18, 19]. The exact mechanisms of the increased incidence of VAP in patients with ABI have still not been properly clarified. Alteration in the level of consciousness and aspiration or micro-aspiration are well known risk factors [20]. Moreover, more severe BI on admission (Glasgow Coma Scale, GCS < 9) has been associated with higher incidence of VAP, presumably due to the need for prolonged MV and sedation [21]. In addition, dysphagia associated with BI is associated with a higher incidence of pneumonia [22]. Furthermore, the systemic inflammatory response in patients with severe BI could predispose them to the development of nosocomial pneumonia [21]. It has indeed been found that ABI-induced immunosuppression and brain–lung interactions may lead to systemic inflammation and pulmonary injury and infection [5]. The same holds for their effects on the reduced phagocytic capability of alveolar macrophages [23]. In addition, studies in BI patients and animal models of ABI indicate that there is massive intracranial production of pro-inflammatory cytokines. As the blood–brain barrier (BBB) is impaired, the release of these cytokines into the systemic circulation is enhanced, which activates inflammatory cascades [24, 25], with subsequent immunosuppression and infection [6]. In addition, specific subgroups of ABI such as patients with TBI, experience acute secondary adrenal insufficiency [26], leading to greater exposure to systemic inflammation and immunοsuppression, with subsequent enhanced incidence of nosocomial infection, and more particularly VAP [27, 28].

Additional risk factors for VAP include younger age, alcohol and drug abuse, barbiturate infusion, smoking, tracheostomy, blood transfusion upon admission, therapeutic hypothermia, and gastric aspiration before intubation [16, 20]. Moreover, factors such as thoracic trauma, omission of the head up position during MV, and less prophylactic antibiotic use have been found to increase the risk of VAP [4, 5, 29]. Chronic lung disease, haemorrhagic transformation and stroke severity on admission were additional risk factors for VAP [30].

### Neurogenic pulmonary oedema

NPO has been defined as the extravasation of protein-rich fluid into the interstitial and alveolar space of the lungs after various pathologies of the central nervous system (e.g. stroke, SAH, subdural haemorrhage, status epilepticus, infections of the central nervous system, and TBI) [31–34]. The diagnosis of NPO is based on the presence of respiratory distress, hypoxaemia, bilateral alveolar opacities with diffuse infiltrates of both lungs, and lack of evidence of left heart failure in the absence of other causes of ARDS [35, 36].

Despite extensive ongoing research, the pathophysiology of NPO is still debated and several theories have been proposed [7, 37]. The most popular theory, however, suggests that an abrupt increase in ICP leads to sympathetic overstimulation and massive release of catecholamines into the systemic circulation, with subsequent generalised vasoconstriction [35, 38, 39]. Systemic vasoconstriction and elevated systemic resistance shift blood from the systemic to the pulmonary circulation. The subsequent increase in hydrostatic pressure in the pulmonary capillaries generates the development of transudative pulmonary oedema and damage to the alveolar capillary barrier. The structural damage of the capillary endothelium results in leakage of protein-rich fluid into the interstitial spaces and alveoli [37–39].

It has been shown that in most patients (i.e., 71%) the onset of the symptoms is acute (< 4 h, 30–60 min after acute neurological injury), although NPO can also be delayed (12–72 h after acute neurological injury) [33, 40]. Risk factors for the occurrence of NPO include older age, more severe BI, delayed treatment and surgery to the vertebral artery [41, 42].

Despite the recent progress in understanding the pathophysiology of NPO, treatment options are limited and mainly supportive. The prognosis is generally poor, with mortality rates ranging between 60 and 100% [43].

### Acute respiratory distress syndrome

ARDS is a life-threating form of acute respiratory failure, characterized by a combination of refractory hypoxaemia and stiff lungs following an initial insult [44, 45]. According to the 2012 Berlin definition, ARDS is defined as severe hypoxaemia of acute onset—established within a week of a known clinical insult or worsening respiratory symptoms not fully explained by lung oedema, with bilateral lung infiltrates on chest X-ray or CT scan [46].

Acute lung injury (ALI)/ARDS is common in BI patients [31, 47], with a reported incidence of between 5 and 30%, depending on the specific types of BI and the inclusion criteria adopted by various studies [7]. A prospective study enrolled 192 patients with a range of neurological disorders and reported an incidence of ALI/ARDS of 35% [48]. For patients with GCS less than 9, the reported incidences were between 20 and 30% [13, 49–51]. ALI and ARDS have been reported in 15–40% of patients suffering from an SAH [52], while the equivalent incidence for isolated TBI has been found to be about 20–25% [31, 47].

The pathophysiological processes of ABI-associated ARDS are complex and are discussed in detail in the Pathophysiology Section. However, experimental and clinical studies suggest that there are differences in the inflammatory pathways between ARDS of neurogenic origin and non-neurogenic ARDS [31, 47]. As previously discussed, ABI induces a systemic inflammatory response, with pulmonary infiltration of neutrophils, cytokine release and immunosuppression, thus increasing the risk of infections, and more particularly pneumonia [5, 21–23, 53]. Moreover, sympathetic hyperstimulation induces fulminant release of catecholamines into the systemic circulation that leads to NPO [37–39], a distinct entity from ARDS [10]. In addition, experimental evidence suggests that intracranial hypertension increases the levels of extravascular lung water amount in poorly aerated lung areas and might directly enhance lung inflammation [54]. All of these mechanisms contribute to the development of ALI/ARDS in patients with ABI and previously healthy lungs, and should be taken into consideration when applying neurocritical care interventions such as MV, which can induce an inflammatory response [10].

Development of ALI in patients with ABI is associated with a threefold increase in mortality and severe residual neurological dysfunction [13]. In patients with ABI, the severity of a neurological event may be characterized by initial GCS and abnormalities in initial brain computer tomographs and has been identified as a risk factor for the development of ALI/ARDS [49, 50]. Moreover, administration of vasoactive agents, a history of drug abuse, and hypertension have been described as additional risk parameters for the development of acute respiratory failure [7, 47]. Finally, predictors of the development of ALI include older age, cardiac arrest, heart failure, chronic obstructive pulmonary disease, cardiovascular, renal or haematological dysfunction [55], sepsis, shock, transfusions, and pneumonia [52].

ALI in BI patients has been reported to have two peaks, an early peak on days 2–3 after the initiation of MV, and a second peak on days 7–8 [56].

## Pathophysiology of lung injuries in patients with isolated brain pathology

**Fig. 2:**
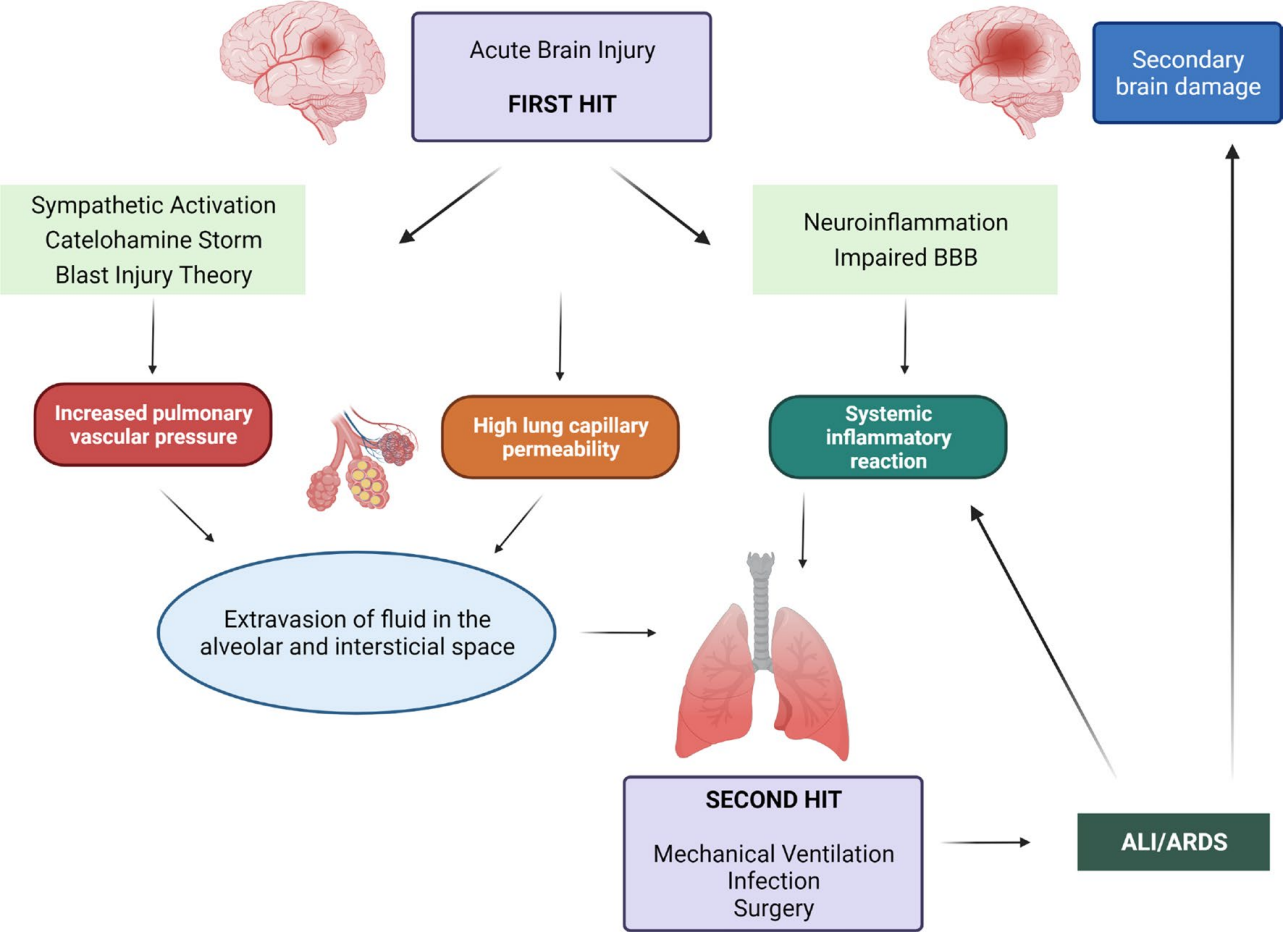
Pathophysiology of lung injury in patients with isolated acute brain injury. ALI, Acute Lung Injury; ARDS, Acute Respiratory Distress Syndrome

The development of pulmonary complications shortly after initiation of BI has been found in several clinical and experimental studies and is well-recognized. Although most clinical and experimental data support the existence of a strong interaction between brain and lungs [57], the pathophysiology of lung injuries in BI patients is still under discussion. Various mechanisms have been described, including neuroinflammation, neurotransmitter-mediated injury, NPO, and adverse side effects of therapeutic management [58, 59] (Fig. 2).

### Sympathetic activation and blast theory

ABI is an acute biomechanical process which develops over time. In the initial stage, the increased sympathetic activity due to the increase in ICP leads to massive catecholamine release and probably the development of NPO [38, 60]. This catecholamine storm leads to rapid and massive blood transfer from the systemic to the pulmonary circulation, resulting in extravasation of fluid in the alveolar and interstitial space due to hydrostatic pressure [60].

The “blast injury” theory is the most widely accepted of the several theories that have been suggested to explain the pathophysiology of NPO [60]. The “blast injury” theory suggests that there is a transient increase in intravascular pressure due to the catecholamine storm following ABI. This increase in intravascular pressure damages the alveolo-capillary membrane, thus leading to NPO [60]. This hypothesis is consistent with the low pulmonary/protein ratio [61]. However, an experimental study of intracranial hypertension has found that there is accumulation of extravascular pulmonary protein and indicated that permeability is high [62]. The pivotal role of sympathetic discharge in the pathogenesis of NPO is further supported by an experimental study that found that haemodynamic changes are responsible for these phenomena. Moreover, elimination of a hypertensive response in brain damaged rats by pre-treatment with α-adrenergic antagonists preserved the integrity of the capillary-alveolar membrane [63]. Additionally, current knowledge suggests that the early use of beta-blockade in patients with severe TBI decreases in-hospital mortality and improves the functional outcome up to 6 months following injury [64]. Although the combination of hydrostatic forces and impaired permeability plays a role in the pathogenesis of NPO, it cannot explain the extravasation of red blood cells in the bronchoalveolar lavage fluid [31, 47]. It has been suggested that capillary hypertension seems to be involved in the complex pathophysiology of NPO [65].

In contrast, some case reports have reported that NPO develops in BI patients without haemodynamic instability, and this suggests that the trigger may be isolated pulmonary vasoconstriction, modulated by catecholamine storm shortly after BI [66]. This is further supported by several experimental studies using α-adrenergic receptor antagonists, as these indicate that inactivation of α-adrenergic receptors of the pulmonary vessels may prevent the development of NPO [67]. Furthermore, Peterson et al. [68] conducted a study on anaesthetised sheep with progressively increased ICP after treatment with α-adrenergic antagonists. The authors reported that NPO was prevented, without severe effects on systemic haemodynamics, as is consistent with isolated pulmonary adrenergic activation [68].

### Double hit theory

In addition to the “blast theory” and the “pulmonary venule adrenergic hypersensitivity” theories, a systemic inflammatory response seems to play an integral role in the pathogenesis of pulmonary injury in patients with ALI [67]. Clinical and experimental studies in BI patients and animal models of ABI indicate that there is a massive cellular biochemical cascade, with intracranial production of pro-inflammatory cytokines. Due to the impaired BBB, the release of these cytokines into the systemic circulation is enhanced, leading to the activation of inflammatory cascades [24, 69]. Microglia and astrocytes are probably involved in the intracranial production of pro-inflammatory cytokines [1]. Microglia, the resident brain macrophages, are morphologically and functionally activated shortly after BI [70, 71] and produce a variety of proinflammatory molecules, including interleukin (IL)-1, IL-6, IL-8, and tumour necrosis factor (TNF)-α [24]. In addition, microglial cell activation plays a crucial role in the alteration of the BBB—by allowing the release of the mediators into the systemic circulation and infiltration of circulated leucocytes into the brain [72, 73]. These phenomena could, therefore, explain the extracranial organ dysfunctions seen in patients with isolated BI [74]. This suggestion is in accordance with the study of Fisher et al. (1999), who reported elevated concentrations of cytokines in the bronchoalveolar lavage of patients with severe BI [75]. In support of these findings, a later published study reported that donor lungs with high concentrations of IL-8 taken from brain dead patients were associated with graft dysfunction, early recipient mortality, and poor prognosis after lung transplantation [76].

These findings are further supported by several experimental studies. Kalsotra et al. reported marked migration of inflammatory cells into the airways and alveolar spaces 24 h after initiation of BI in animal models. This was accompanied by major enhancement of the production of pulmonary leukotriene B_4_ [77]. In an experimental study, Campbell et al. showed that intracranial administration of IL-1β increases hepatic production of chemokines, followed by elevation in neutrophil levels in the brain, liver, and blood [78]. An additional study in animals with experimentally induced intracerebral haemorrhage supported the hypothesis that ABI is associated with significant neuroinflammation, with marked expression of intracellular adhesion molecule (ICAM)-1 and tissue factor in both brain and lungs. Wu et al. noted that pulmonary expression of these mediators was associated with morphological alterations in the lungs [79]. In a similar manner, in an experimental model of SAH, the lungs exhibited significant expression of ICAM-1, vascular cell adhesion molecule (VCAM)-1, and E-selectin [80].

Thus, several clinical and experimental studies in stroke patients and animal models support the hypothesis of stroke-induced immune suppression [4, 5]. The activation of the immune system in stroke is also characterized by two peaks. The first peak is early transient activation [81, 82], followed by a later second peak from systemic immune suppression [82]; these immune responses include a rapid decrease in peripheral blood lymphocytes and functional deactivation of monocytes [83]. It has also been reported that the catecholamine storm associated with ABI may correlate with lymphopenia [84]. This pathogenetic mechanism is also supported by an experimental study in post-stroke mice, which found that ß- antagonists reduced the incidence of bacterial complications, which may be evidence that catecholamines have an integral role in the pathogenesis of immunosuppression [84]. The same study found that interferon (IFN)-γ production was compromised and that the natural killer and T-cell responses were reduced—leading to failure in antibacterial defense and to bacterial infections [84].

In conclusion, the systemic effects of inflammatory mediators in patients with BI create a systemic inflammatory environment, and the “first hit” makes extracranial organs vulnerable to secondary procedures that enhance inflammation, such as MV, surgery and infections, that is, the “second hit”[7].

## Ventilatory strategies in acute brain injury: What is different?

MV is a lifesaving tool in therapeutic management, and is frequently performed in BI patients [4, 5]. Despite being lifesaving, MV can exacerbate pulmonary and systemic inflammation, thus leading to ALI [1]. Furthermore, it has been established that traditional MV with high tidal volumes (VT) is an independent risk factor for ALI in critically ill BI patients [85]. Thus, MV could play a key role in the occurrence of acute respiratory failure in BI patients [10].

The pathogenetic mechanisms include overstretching, repeated alveolar collapse, and re-expansion in each breath [86]. Moreover, ventilator-associated lung injury could be triggered by the transformation of mechanical to biological stimuli in the lung [87]. The result is a deleterious inflammatory cascade, which is associated with local tissue injury and potential spread to extrapulmonary organs and systems, a process that is often associated with multi-organ failure [88].

On the other hand and as mentioned above, respiratory insufficiency is a common complication in critically ill BI patients. Despite the lack of evidence on the management of patients with both ABI and respiratory failure, it is clear that the ventilator strategy should be doubly protective for the lungs and the brain.

Hypoxia is common in neurocritical care patients, and it is well-established that partial arterial oxygen tension of 58 mmHg or SpO_2_ below 90% in the first few hours after initiation of the BI is associated with a twofold risk of mortality [89]. In addition, such a level of hypoxaemia could lead to decreased cerebral oxygen delivery, which ultimately could cause intracranial hypertension due to hypoxaemia-mediated vasodilation [90].

Recent guidelines from the European Society of Intensive Care Medicine [12] on the MV for patients with ABI propose that the optimal target range of PaO_2_ should be between 80 and 120 mmHg [91].

### Tidal volume

Although it is well-documented that protective MV—with low VT and positive end-expiratory pressure (PEEP)—decreases mortality in patients with ARDS, it is still unclear whether this ventilator strategy should be extended to all critically ill patients [92], and it should be borne in mind that the protective ventilator strategy may lead to self-inflicted lung injury [92] and hypercapnia [85]. In particular in BI patients, modest increases in PaCO_2_ are associated with cerebral vasodilation, resulting in intracranial hypertension, and higher cerebral blood volume [10, 85]. On the other hand, BI patients are traditionally ventilated with high VT, on the basis of the observation that hypocapnia reduces ICP, and in order to maintain normal ICP [10]. However, hyperventilation and the resulting hypocapnia can be detrimental for BI patients, especially during the first 24 h after the initiation of the event, when cerebral homeostasis is critically impaired [93, 94]. As previously discussed, MV with high VT could induce further brain and lung injury (i.e., “second hit”) and extracranial organ failure [7]. It is unfortunate that—due to the different pathophysiological mechanisms of ALI and safety issues—most important trials of lung protective ventilation exclude patients with ABI. Nevertheless, some studies have reported that ventilation with low VT achieves better neurophysiological protection and that this is associated with a lower incidence of ALI in critically ill neurological patients [13, 95, 96], although it still debated whether the protective ventilation strategy should be extrapolated to the prehospital and emergency environment during the acute resuscitative phase (12–24 h) [97]. Despite the lack of robust evidence, the recent recommendations of the European Society of ICM state that there is a consensus that the optimal range of PaCO_2_ lies between 35–45 mmHg [91]. Protective MV with VT of 6–8 ml/kg can help to avoid ALI [4, 5, 10, 91].

### PEEP

PEEP is part of the protective ventilation strategy to improve oxygenation and lung compliance. PEEP can not only prevent alveolar collapse, but also recruit collapsed alveoli. This then improves brain microcirculation, and finally reduces atelectasis [1, 4, 5, 88, 98]. However, in BI patients, PEEP may also alter CBF, reduce cerebral venous return, and increase ICP [85, 99, 100]. The mechanisms of ICP-elevation using PEEP are complex and involve many factors, including intracranial and intrathoracic compliance, systemic haemodynamic parameters, presence of hypovolemia and cerebral autoregulation [1, 4, 5, 10]. Observational studies have demonstrated that high PEEP in patients with BI lead to reductions in cerebral perfusion pressure (CPP) and CBF- due to impaired haemodynamic parameters of the systemic circulation and especially mean arterial pressure (MAP) [101, 102]. However, it has been suggested that the application of PEEP is only associated with increased ICP when PEEP causes alveolar hyperinflation. In contrast, when PEEP determines alveolar recruitment with reduction or no change in PaCO_2_, there is no effect on cerebral perfusion or ICP [7]. On the other hand, even if PEEP remains stable, it may cause an increase in intrathoracic pressure, and thus reduce MAP, venous return, and ICP [103]. Maintenance of euvolemia, by using hypertonic solutions in particular, could probably minimize the effects of PEEP on CPP and MAP [100].

Given the complex pathophysiological interactions between lungs and brain in critically ill patients with neurological injury, advanced monitoring that includes invasive ICP measurements, oxygen jugular saturation, and the partial oxygen pressure of brain tissue is recommended to optimize ventilation strategy and cerebral oxygen delivery in patients with ABI [104].

Even though the various causes of BI appear to coalesce in common pathogenetic mechanisms [22], specific recommendations and evidence should be considered for each specific subpopulation, in order to minimize the risk of pulmonary complications and cerebral dysfunction [11]. The current consensus is that neurocritical patients without lung injury may benefit from a protective ventilation strategy for the lungs, using lower VT and moderate levels of PEEP. However, intensive multimodal monitoring is of major significance, in order to ensure cerebral and systemic haemodynamics.

### Spontaneous breathing mechanical ventilation in acute brain injury

Patients with severe BI are often admitted in the ICUs for neuromonitoring and MV [105]. Sedation and analgesia are frequently mandatory and have specific roles following ABI [106]. They are used for several reasons: to control anxiety and motoric unrest, pain and agitation, avoid autonomic disability, control ICP, reduce brain metabolism, and optimize MV [106, 107]. In the general adult and paediatric ICU patients, light rather than deep sedation is recommended, for mechanically ventilated patients, in order to shorten the duration of MV and length of hospital stay [108, 109]. Unfortunately, there is little evidence for neurocritical care patients, because BI patients are often excluded in these studies [110, 111]. On the other hand, brief cessation of sedation for daily wake-up tests may be beneficial to critically ill BI patients, by allowing clinical neuromonitoring, the detection of early warning neurological signs and neuroanatomical localization of pathology, and by helping to guide appropriate therapy [112, 113]. Daily neurological assessments may be able to reduce the duration of MV and the need for tracheostomy [114]. However, withdrawal of sedation may result in significant activation of the sympathetic autonomic system, with deterioration in cerebral haemodynamics [113], so that the benefits of daily neurological assessments must be weighed against the associated risks.

In contrast to non-neurocritical care patients, BI patients usually do not have the primary respiratory indication for ventilator support [115, 116]. Moreover, BI patients are subject to prolonged MV and delayed extubation [87, 117], despite the fact that they are often able to breathe spontaneously [115, 116].

However, while interest in the use of partially supported breathing modes is increasing [118, 119], the role of spontaneous breathing [120] ventilation in patients with ABI is less well-established [121]. Spontaneous respiratory effort has been shown to be beneficial by improving gas exchange and oxygenation, haemodynamics, and non-pulmonary organ function [10, 106, 122–124]. Moreover, SB is associated with reduced sedation [125], thus facilitating daily neurological assessment. In addition, SB seems to prevent diaphragmatic dysfunction by allowing diaphragmatic muscle contractions [126], improving ventilation-perfusion matching, recruiting the lungs [123] and reducing dead space [127]. Thus, many neurocritical care physicians allow light sedation, if this is tolerated by the patient.

On the other hand, there is accumulating evidence that SB may cause or even worsen ALI [123]. SB contributes to the transpulmonary pressure, thus resulting in a proportional increase in VT [123]. Moreover, SB exertion may also increase transvascular pulmonary pressure, thus leading to pulmonary oedema and VILI [123, 128]. Furthermore, the “pendelluft” phenomenon during SB-MV may contribute to ALI by overstretching the dependent lung areas [122]. Finally, asynchrony between patient and ventilator can exacerbate ALI and is associated with prolonged MV and increased mortality [14, 129]. “Double triggering” is one of the most common forms of asynchrony between the patient’s exertions and the ventilator [129, 130] and may result in large VTs with injurious effects [129, 131].

In summary, even though there are no clinical studies in patients with ABI, experimental data, preliminary results of a clinical study and observational findings in patients with ALI/ARDS suggest that SB-ventilation can be used without undue harm [122]. As the use of VT as a surrogate marker for lung distension and inspiratory exertion has limitations, there is an urgent need to find new methods to establish the safety of SB ventilation [132]. Reliable assessment of respiratory drive and inspiratory effort is essential to estimate the balance between beneficial and deleterious consequences of SB during the MV of BI patients [122, 128, 132].

## Conclusion

During the last decade, it has been demonstrated by experimental and clinical studies that ABI can cause severe dysfunction of peripheral extracranial organs and systems. The aim of the current review has been to focus on ALI occurring shortly after BI. As we have shown, severe BI induces autonomic dysfunction and a severe systemic inflammatory response, so that the lungs of the patients are vulnerable to secondary inflammatory stimuli. A strategy for MV could help to modify inflammatory events and thus alleviate further damage to the brain and lungs. Further studies are needed on the complex pathophysiological interactions between brain and lungs in patients with isolated BI.

## Data Availability

Not applicable.
